# Development of a deep learning method to identify acute ischaemic stroke lesions on brain CT

**DOI:** 10.1136/svn-2024-003372

**Published:** 2024-11-20

**Authors:** Alessandro Fontanella, Wenwen Li, Grant Mair, Antreas Antoniou, Eleanor Platt, Paul Armitage, Emanuele Trucco, Joanna M Wardlaw, Amos Storkey

**Affiliations:** 1 The University of Edinburgh School of Informatics, Edinburgh, UK; 2 The University of Edinburgh Centre for Clinical Brain Sciences, Edinburgh, UK; 3 The University of Sheffield School of Medicine and Biomedical Sciences, Sheffield, Sheffield, UK; 4 University of Dundee School of Science and Engineering, Dundee, UK; 5 UK Dementia Research Institute, Edinburgh, UK

**Keywords:** Stroke, CT, Brain

## Abstract

**Background:**

CT is commonly used to image patients with ischaemic stroke but radiologist interpretation may be delayed. Machine learning techniques can provide rapid automated CT assessment but are usually developed from annotated images which necessarily limits the size and representation of development data sets. We aimed to develop a deep learning (DL) method using CT brain scans that were labelled but not annotated for the presence of ischaemic lesions.

**Methods:**

We designed a convolutional neural network-based DL algorithm to detect ischaemic lesions on CT. Our algorithm was trained using routinely acquired CT brain scans collected for a large multicentre international trial. These scans had previously been labelled by experts for acute and chronic appearances. We explored the impact of ischaemic lesion features, background brain appearances and timing of CT (baseline or 24–48 hour follow-up) on DL performance.

**Results:**

From 5772 CT scans of 2347 patients (median age 82), 54% had visible ischaemic lesions according to experts. Our DL method achieved 72% accuracy in detecting ischaemic lesions. Detection was better for larger (80% accuracy) or multiple (87% accuracy for two, 100% for three or more) lesions and with follow-up scans (76% accuracy vs 67% at baseline). Chronic brain conditions reduced accuracy, particularly non-stroke lesions and old stroke lesions (32% and 31% error rates, respectively).

**Conclusion:**

DL methods can be designed for ischaemic lesion detection on CT using the vast quantities of routinely collected brain scans without the need for lesion annotation. Ultimately, this should lead to more robust and widely applicable methods.

**WHAT IS ALREADY KNOWN ON THIS TOPIC**

**WHAT THIS STUDY ADDS**

- Our study presents a deep learning (DL) method for detecting ischaemic lesions on CT scans that was developed using a large data set of routinely collected, expert-labelled (but not annotated) brain scans from over 3000 patients within 6 hours of acute stroke onset. By leveraging this extensive data set, we were able to better explore the capabilities of DL for CT interpretation using large numbers of routinely collected scans. The method achieved 72% accuracy in detecting ischaemic lesions, performing better on follow-up scans compared with baseline scans and with better detection of larger lesions compared with smaller ones in alignment with human diagnostic behaviour.

**HOW THIS STUDY MIGHT AFFECT RESEARCH, PRACTICE OR POLICY**

- This approach demonstrates the potential to develop robust and widely applicable DL systems from large numbers of routinely collected scans, better representing real-life patients with natural heterogeneity.
- Such systems could ultimately provide more accurate and timely image interpretation for patients with acute ischaemic stroke, potentially improving treatment decisions and outcomes.

## Introduction

 Non-contrast-enhanced CT is the most commonly used brain imaging modality for stroke assessment in the acute setting due to its availability and speed.^1^ While brain CT in this context is primarily used to identify haemorrhage and other contraindications to thrombolytic therapy (eg, structural stroke mimics such as brain tumour) rather than to identify ischaemia, positive detection of an ischaemic lesion confirms the diagnosis and may improve implementation of thrombolysis and thrombectomy treatment pathways. Accurate identification of ischaemic features on CT can be challenging and depends on the reviewing clinicians experience (eg, stroke clinician vs radiologist vs trainees)^2^ and the scan timing (ischaemic lesions become more visible with time). Computer-aided diagnosis may reduce delays, improve consistency of image interpretation^3^ and increase treatment success.^4^ However, current techniques are still in development.^5^ While there are several commercially available systems that predict features or provide clinical scores from a brain CT scan for stroke^6^ such as the Alberta Stroke Programme Early CT Score (ASPECTS),^7^ to the best of our knowledge these systems were developed using annotated images which (due to the effort required to produce these annotations, ie, to draw round the lesions) necessarily limits the size (and representativeness) of the imaging data set used for development. In addition, the precision of such annotations is not known but must directly affect the quality of future lesion detection using the system.

In this study, we aim to develop a deep learning (DL) method for acute ischaemic stroke lesion diagnosis using a large data set of routinely collected brain CT scans from an international multicentre clinical trial where expert readers have labelled the scans for ischaemic lesion presence, location and extent (and for various other acute and chronic brain features), without annotations. In other words, rather than indicating exactly what to look for in images, we allow the DL methodology to independently assess thousands of features within scans that may correctly indicate the presence of an ischaemic lesion. We also explore the interpretability of our model, the impact of different ischaemic lesion sizes and background conditions on its performance and quantify its agreement with the assessment of expert radiologists.

## Methods

### Data source and expert labelling of imaging data

We used CT data from the Third International Stroke Trial (IST-3)^8 9^ which was a randomised controlled trial of intravenous alteplase for patients with acute ischaemic stroke. The study recruited 3035 patients and baseline CT brain imaging was acquired within 6 hours of stroke onset followed by a 24–48 hour follow-up CT for most patients (those that survived and were well enough to be reimaged). All patients recruited in IST-3 were screened by experts using all available data including imaging to confirm the presence of genuine ischaemic stroke and to exclude haemorrhage or stroke mimics.

The IST-3 imaging data set consists of raw CT data in DICOM (Digital Imaging and Communications in Medicine) format which were obtained from 156 different hospitals in 12 countries worldwide. The recruiting hospitals were instructed to submit all relevant imaging for each patient acquired according to their own stroke imaging protocols with only minimal basic requirements imposed by the trial. Therefore, the IST-3 CT data set is similar to the imaging acquired during routine clinical care.

All brain scans were centrally assessed by a single expert drawn from a panel of 10 and who had undergone prior assessment for consistency (inter-rater agreement>kappa 0.7^8^). The experts were masked to all other data except whether scans were acquired at baseline or follow-up. They provided labelling for a range of acute and chronic brain changes related to stroke including acute ischaemic brain lesions,^10 11^ acute arterial obstruction (on non-enhanced CT, presence of a hyperattenuating artery^12^), and at follow-up acute haemorrhage, all quantified by location and extent (1–4 with 1 being smallest and 4 the largest) using clinically validated methods. In particular, the algorithm used to classify the different lesions can be found in Appendix 5 of the IST-3 data set description (https://datashare.ed.ac.uk/bitstream/handle/10283/1931/DescriptionofIST3SharedDatasetAugust2015.pdf) . The IST-3 has developed a comprehensive algorithm for coding lesion location and size, widely used in acute stroke trials which takes into account various factors such as the affected brain regions, infarct type and extent. The schema identifies the vascular territory and extent of involved tissue using hierarchical numbers and reflects typical patterns of infarcts commonly seen in acute ischaemic stroke. The method aligns with other commonly used visual scoring systems such as ASPECTS,^2^ although has the advantage of classifying all vascular territories (not just the middle cerebral artery (MCA)) indicating the location and extent of the lesion (not just the extent) and reflecting the likely site of arterial occlusion. The numbers reflect the relative extent of the affected arterial territory or combinations of territories but do not correspond to absolute volumes of tissue. Ischaemic brain lesion location was divided into seven anatomical categories relating to arterial blood supply and lesion type: Major arterial territories of cerebral hemispheres (three categories—anterior, middle and posterior cerebral—ACA, MCA and PCA, respectively), cerebral border zones (one category), posterior circulation (two categories) and lacunar (one category). The experts also assessed and labelled scans for chronic brain changes,^13^ such as atrophy, leukoaraiosis, old stroke lesions and other benign incidental abnormalities which may impact the expert or DL assessment of the imaging. All expert assessment was stored separately for imaging as text, that is, the presence and location of abnormalities were not annotated directly on imaging, see the online supplemental figure 1.

### Preprocessing of CT scans

We previously developed a pipeline to clean and preprocess clinical CT data for DL development.^14^ The pipeline included several preprocessing steps such as identifying axial images, converting DICOM data to Neuroimaging Informatics Technology Initiative format,^15^ removing localisers and poor-quality scans, cropping redundant space and normalising image brightness. To account for varying slice numbers, a uniform sampling approach was applied, selecting 11 slices from each scan. The processed scans were standardised to the dimensions of 500×400×11 (height, width and slice number).

### DL method

Our goal was to classify CT brain scans as either having an ischaemic lesion (positive) or not (negative) and, if positive, to predict which side of the brain is affected (left, right or both). To study the impact of lesion location on the accuracy of the model, we also compared the performance of our method across different regions of the brain.

To achieve this, we employed PyTorch to design a DL method using a multitask learning (MTL) convolutional neural network (CNN) with two heads and seven convolutional layers. We divided our data set into training, validation and test sets using a 70-15-15 split with all the scans of each patient appearing in only one data set.

**Figure 1:**
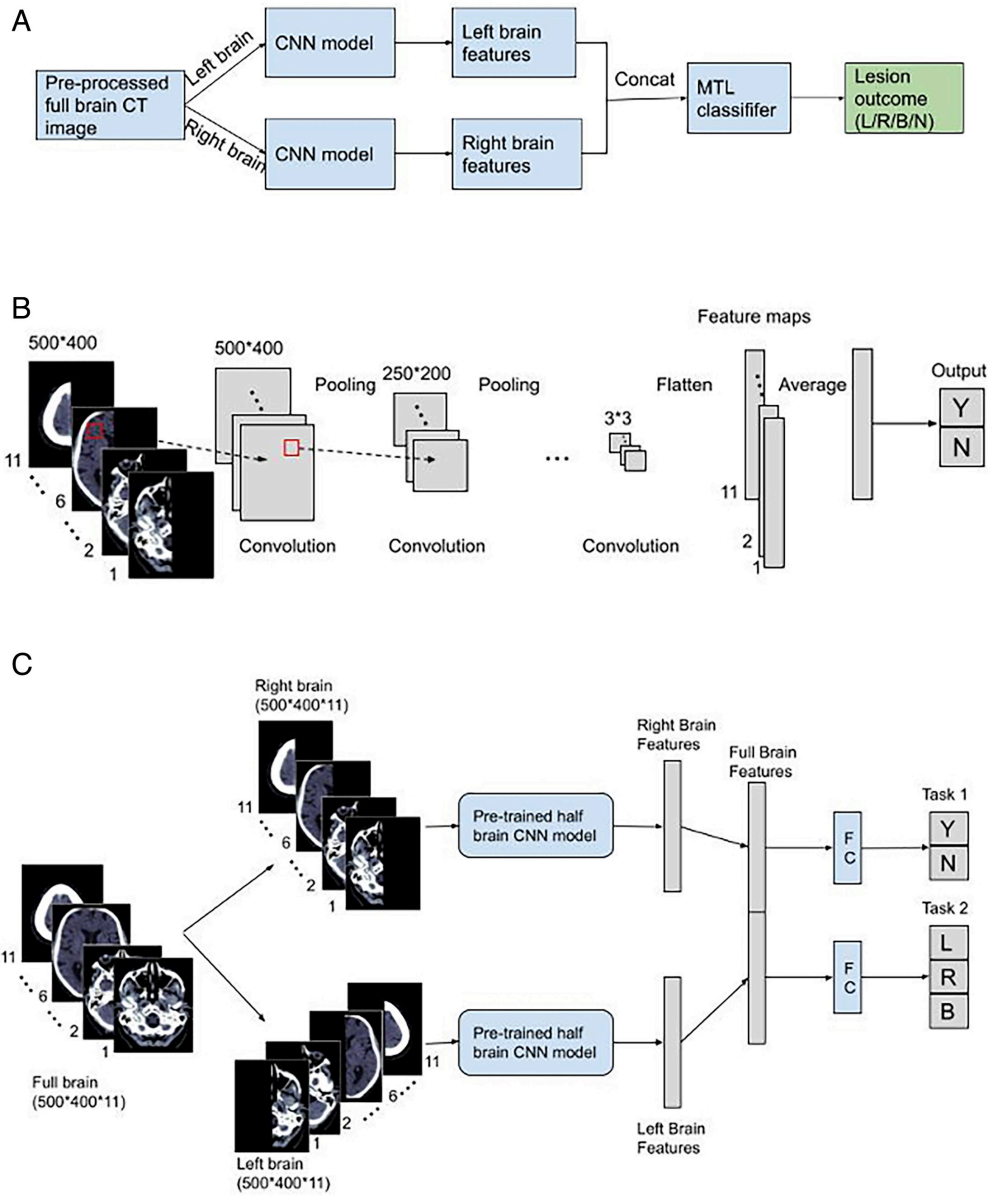
Multitask deep learning method logic (A), half brain CNN model architecture (lesion outcomes are left, right, both, none) (B) and multitask learning (MTL) architecture (C). FC indicates fully connected layers. CNN, convolutional neural network.

We trained the algorithm to learn acute lesion features from each side of the brain separately. To accomplish this, we split all scans into two halves at the sagittal midline creating half-brain input. We then concatenated the extracted features from each side into a full-brain lesion feature vector which was used by a multitask classifier to predict lesion presence (Task 1) and, if positive, the side of the brain affected (Task 2). The logic of our MTL architecture is depicted in figure 1A.

In the first stage of training, to help prevent confounders, our model takes half brain inputs and is solely trained to classify if a lesion is present or absent. Each layer of the CNN performs two-dimensional convolution, batch normalisation and average pooling on each slice. At the end of the seventh layer, we average each feature map across all 11 slices. The architecture of the 7-layer CNN model is illustrated in figure 1B.

In the second stage, we add a classifier with two headers, each comprising of one fully connected layer and one output layer for the corresponding task. The complete architecture of our method is shown in figure 1C. In particular, we first trained the half-brain model on its own and then fine-tuned the whole architecture. The hyperparameters employed are listed in the online supplemental table 1.

In the first stage of training, we focus on learning features of acute lesions independently from each side of the brain. By separating the learning of features from each side of the brain, we ensured that the model could develop distinct representations of lesions that may manifest differently depending on their location. By isolating the feature extraction process for each hemisphere, we minimise potential interference from the other side of the brain. Once the model demonstrates proficiency in this task, we combine the features from both sides into a unified lesion feature vector that represents the entire brain. This approach allows the model to effectively encapsulate global brain information while maintaining sensitivity to localised patterns.

Furthermore, by processing each slice individually during the first stage, we enhance the model’s sensitivity to subtle variations that may appear in different slices. By using two-dimensional convolutions, we efficiently capture localised features without the computational complexity of three-dimensional convolutions. This not only simplifies the architecture but also improves computational efficiency, allowing for a more efficient integration of information across slices.

### Comparison with existing methods

Although we did not find any publicly available open-source methods specifically tailored for stroke lesion detection from brain CT scans, we benchmarked our approach against several architectures commonly used in computer vision: Vision Transformer (VIT), Swin Transformer, ResNet-18 and ResNet-50.

For the VIT, we used six transformer blocks, each with 16 heads in the multihead attention layers and a patch size of 25. For the Swin Transformer, we employed four transformer blocks with 3, 6, 12 and 24 attention heads, respectively, a window size of 7 and a patch size of 4.

### Agreement between DL classification and expert readings

The accuracy and reliability of CT scan labelling can be influenced by the quality of the data and the experience of the clinicians. A previous reliability study^16^ compared the assessments of seven of our expert contributors for CT and concurrent CT angiography (CTA) scans from 15 patients. The study showed substantial agreement between these experts as measured by Krippendorff’s alpha (K-alpha) coefficient with bootstrapping.

To assess the agreement between our DL algorithm and the expert readings, we used 14 of the same 15 patient scans. One scan was excluded due to comprising two image sets, one through the skull base and one through the skull vault. To ensure fairness, we withheld the CT scans of these 14 patients from the training and validation data sets used to develop our DL method.

### Model interpretability and explanation

To gain insights into the factors driving the predictions of our DL model, we employed counterfactuals, a method for generating explanations for model outputs. Counterfactual explanations identify how an input image should be modified to produce a different prediction enabling us to identify the most important features in the image for the classification outcome.^17^ To accomplish this, we employed the method described by Cohen *et al*^18^ later referred to as ‘gifsplanation’.

**Figure 2:**
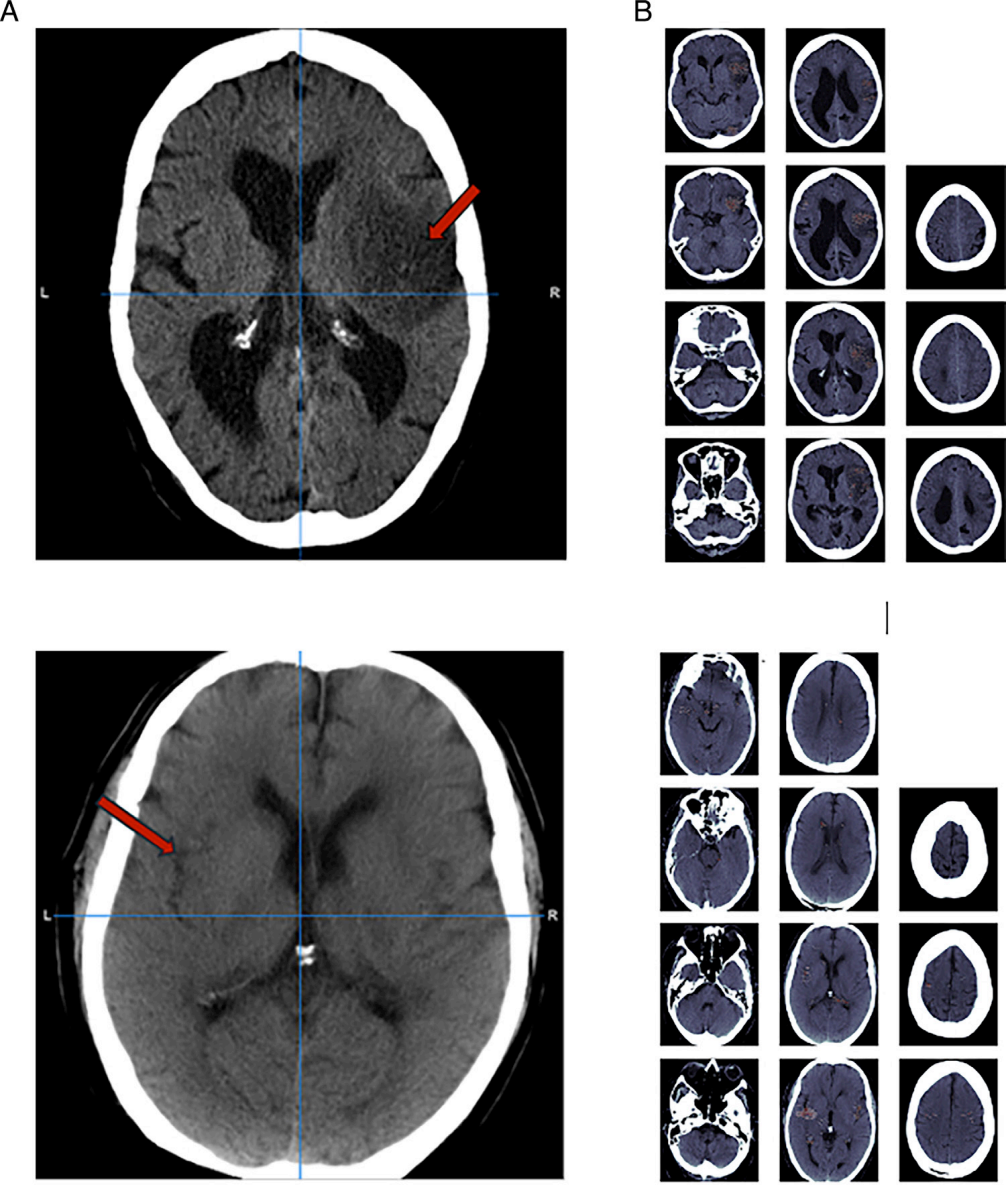
Image with a clear lesion in the right MCA region (A) and corresponding saliency maps highlighting the lesion in red (B). In (C), the lesion in the left MCA region is less clear and therefore the model is less certain about the lesion location as shown by the corresponding saliency maps in (D). For the saliency maps, the voxels in the 99 percentile are displayed.

In particular, we considered an image with an ischaemic lesion and reduced the probability of a lesion to less than 0.01. By considering the difference between the original image and the counterfactual image, we obtained an attribution map of the most salient regions. Intuitively, the voxels that are more affected by the class change are the ones encoding more class-specific information and therefore relevant for lesion detection. Examples are shown in figure 2.

## Results

### Data

Out of the 3035 patients enrolled in the IST-3 study, the majority (95%) underwent CT scans while the remaining had MRI scans. Some CT data was corrupted due to long-term storage in the DICOM format. Consequently, only scans from 2578 patients (85%) were successfully retrieved from our DICOM server. These patients had a total of 10 659 CT image sets. Among these sets, there was significant variability, including non-axial orientations (18%), localisers (5%), bone reformats (6%), separated skull base/vault (12%) and scans with poor patient positioning leading to registration failure (4%). After these processing steps, a total of 5772 image sets from 2347 patients (1243 women and 1104 men) were selected. The median age of the patients was 82 years (IQR 74–86 years). After excluding the 14 patients reserved for assessing algorithm-expert agreement, 5730 unique scans from 2333 patients were used in subsequent analyses.

**Figure 3:**
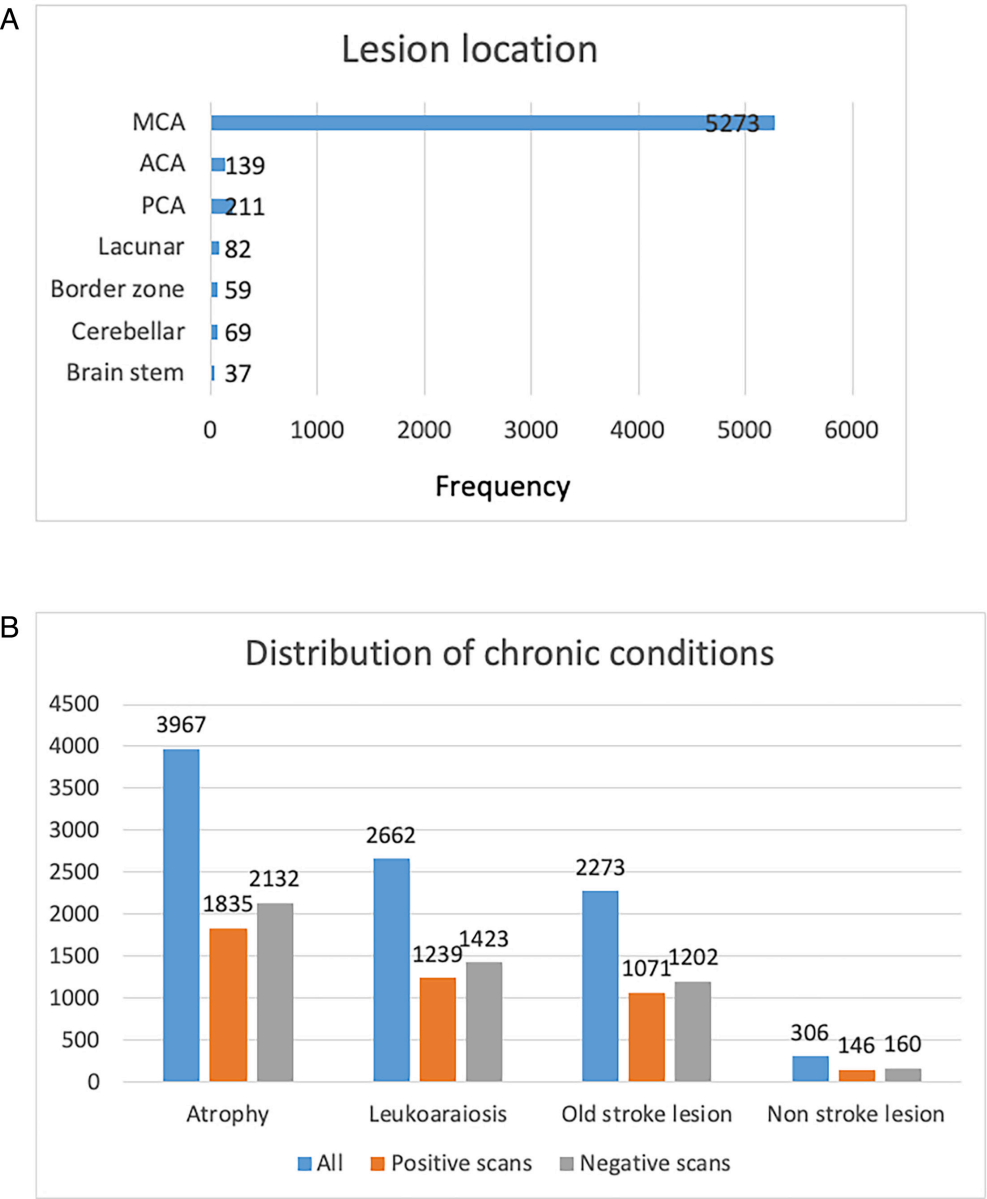
Lesion location (**a**) (MCA, ACA, PCA=middle, anterior and posterior cerebral arteries, respectively) and chronic condition, (**b**) distribution on the processed IST-3 data set subdivided for scans that were positive/negative for the presence of an ischaemic lesion.

The data set was split into three sets: 4031 scans from 1633 patients for training, 844 scans from 350 patients for validation and 855 scans from 350 patients for testing. Of the 5772 total CT scans, approximately 54% (3102 scans) were positive for an ischaemic lesion according to experts. Of the positive scans, 54% (1667 scans) showed lesions on the left side of the brain, 45% (1386 scans) showed lesions on the right side and the remaining (49 scans) showed lesions on both sides of the brain. However, the distribution of lesion locations was uneven as shown in figure 3A. In addition, 5274 scans were labelled with background or chronic brain conditions with the distribution of these conditions shown in figure 3B.

### Model selection

On the validation data set, we investigated the optimal number of convolutional layers to employ in our model. Online supplemental figure 2, in the supplementary material, displays the accuracy obtained with an increasing number of layers. We can observe an initial performance improvement followed by a plateau after six layers. Therefore, we determined that utilising seven convolutional layers provides a favourable trade-off between performance and computational resources.

### Overall accuracy, precision, specificity of the DL model

The overall accuracy, precision and specificity of the DL model were evaluated using 855 test scans including 416 baseline scans and 439 follow-up scans. The model achieved an accuracy of 72% for classifying a given full brain CT scan into one of four classes: Left-side brain lesion, right-side brain lesion, bilateral lesions or no lesion. The VIT achieved a test accuracy of 58% while the Swin Transformer reached 60%. In comparison, ResNet-18 achieved 64% accuracy and ResNet-50 performed slightly better with 66%.

These results indicate that transformer-based architectures such as VIT and Swin Transformer, achieved lower accuracy in this task which aligns with previous findings that vision transformers often require large training data sets to effectively learn visual representations^19^ and are frequently outperformed by CNNs in medical imaging tasks.^20^ Additionally, the convolutional architectures (ResNet-18 and ResNet-50) tested still performed significantly worse than our custom architecture which achieved an accuracy of 72%.

We did not do any formal comparison with other, for example, commercially available AI diagnostic tools in stroke as part of this analysis but the Brainomix AI stroke diagnosis tool has been previously evaluated on a large stroke CT data set which included some cases from IST-3. The performance which was no better than the results described here is published in Mair *et al.*^21^

The accuracy (76%) on follow-up scans was considerably higher than the accuracy on baseline scans (67%). For Task 1 (classifying an image as positive or negative for an ischaemic lesion), the model achieved an accuracy of 75%. For Task 2 (classifying the side of the ischaemic lesion for scans classified as positive in Task 1), the model achieved an accuracy of 91%.

On the entire test set, the model demonstrated higher specificity (80%) than sensitivity (70%). The sensitivity on follow-up scans was 78% while that on baseline scans was 56%. The specificity of follow-up scans was 83% compared with 79% on baseline scans.

### Accuracy by lesion location

Accuracy within brain regions was evaluated on 409 scans (out of the 416 positive ones) from the test data set which included both lesion side and location labels. Of the 409 images, 148 were baseline and 261 were follow-up scans. Our algorithm demonstrated high accuracy for ischaemic lesions in the ACA region (21/28, 75%), followed by the MCA region (248/363, 68%) and PCA region (18/34, 53%). However, it had lower accuracy for brain stem (1/5, 20%), lacunar (3/9, 33%) and cerebellar (5/15, 33%) lesions (see online supplemental table 2a). It should be noted that these types of lesions were extremely rare in the data set which hindered the generalisation capabilities of our model.

Some patients have multiple lesions affecting different regions. The accuracy of our model increased with an increasing number of ischaemic lesions, as shown in the online supplemental table 2b. On average, scans with only one lesion had a classification accuracy of 62%, scans with two lesions had an accuracy of 87% and scans with more than two lesions had 100% accuracy.

### Different infarct sizes and background conditions

The accuracy of our algorithm varies across different ischaemic lesion sizes. The scans with the largest lesion sizes (3 and 4) and those with no lesion showed the highest accuracy (80%). The scans with lesion sizes 1 and 2 (small and very small) are more difficult to classify resulting in an accuracy of only 49%. We observed a higher accuracy in classifying ischaemic lesions in follow-up scans compared with baseline scans across scans with different lesion sizes (see online supplemental table 2c).

In addition, we found that 779 out of 855 test scans had background brain conditions. Among these scans, non-stroke lesions and old stroke lesions had the worst error rates, at 32% and 31%, respectively, followed by atrophy (28%) and leukoaraiosis (26%) (online supplemental table 2d).

### Reliability compared with human experts

To evaluate the agreement between our model and expert readings, we compared the classifications of our algorithm with those of seven human experts on the same 14 scans. We calculated the k-alpha value of our algorithm’s classification compared with each expert’s reading and found an average value of 0.41 which is lower than the general k-alpha among the experts (0.72) (see online supplemental table 3a). However, as depicted in online supplemental table 3b, there were instances involving two scans (patients 7 and 12) where the consensus among experts diverged from the label present in our data set; this label is regarded as the ground truth by our algorithm and was consequently matched by its predictions. Moreover, the expert agreement data we used was based on an assessment of both CT and corresponding CTA data for each patient whereas our DL method only used the CT images. Indeed, using data from another study,^2^ we also computed the K-alpha value from eight experts each rating the same CT scans (without having access to CTA images) . The K-alpha value for expert agreement in this analysis was lower than the level of agreement obtained when both CT and CTA data were available: 0.51, with a 95% CI of (0.46, 0.57).

### Saliency maps evaluation

Sample saliency maps are shown in figure 2 for scans with lesions in the MCA region of the brain. For scans with a lesion that is easily distinguishable, the saliency maps usually highlight the relevant brain areas (figure 2A,B). In cases where the lesions are less clear, the areas highlighted by the saliency maps are more scattered, a sign the model is less certain about the lesion location while nevertheless usually still highlighting the correct region (figure 2C,D). A quantitative evaluation of the saliency maps is presented in the online supplemental material.

## Discussion

In this study, we developed a multitask DL algorithm capable of detecting ischaemic lesions of any type and in any brain location using 5772 CT brain scans collected from patients who had stroke and labelled but not annotated for lesion location/extent. Our best-performing method achieved an accuracy of 72% in correctly detecting ischaemic lesions and performed better on follow-up scans compared with baseline scans which is consistent with human performance.

We also investigated the impact of lesion location, lesion type, lesion size and background brain changes on the performance of our DL system. However, training a DL model requires a large number of examples.^22 23^ In our study, the distribution and type of ischaemic lesions commonly encountered were highly skewed with most cases showing lesions caused by large-medium vessel occlusion affecting the MCA territory of the brain. As a result, our algorithm was less successful in detecting less frequently occurring lesions such as brain stem lesions, lacunar lesions and cerebellar lesions which had fewer example cases. Furthermore, some ischaemic lesions are much smaller than others affecting the performance of our model.

We also analysed four types of background brain changes and found that our DL system had the highest classification error for scans with old stroke lesions and scans with other lesion types not related to stroke. However, a balanced data set where each feature is represented equally would be required to determine the importance of DL system confounding by specific acute lesions or background brain changes. Further studies in the future are needed to address this issue.

The average agreement between our algorithm and seven experts was relatively low compared with the agreement among the seven experts. There are likely multiple reasons for this. First, ground truth is not always obtainable in medical imaging and our analysis was based on a clinical gold standard reference that was qualitatively assessed by a single expert which is known to be imperfect and influenced heavily by clinician experience. In other words, our DL system learnt from the best available data but the data were imperfect. Second, the expert agreement data we used included both CT and corresponding CTA data for each patient while our DL method only used the CT images. The addition of CTA makes it more likely for our experts to reach the correct answer (and thus agree) for each scan. In fact, using data from a separate analysis, we observed lower agreement among experts when only CT images were provided which was more similar to our expert-DL agreement.

Early detection of ischaemic stroke is important for improving patient outcomes due to the time-sensitive nature of available treatments. Accurate early detection influences several aspects of acute stroke management such as appropriate patient prioritisation in emergency settings, selection for treatment with thrombolysis and/or thrombectomy and the early initiation of secondary prevention measures which can reduce the risk of recurrent strokes.

However, despite its importance, early detection of ischaemic stroke presents several challenges such as the subtle presentation of early-stage lesions and the presence of stroke mimics and chameleons as demonstrated in a previous analysis of a commercially available tool which revealed many shortcomings.^21^

The superior performance of our model on follow-up scans compared with baseline scans aligns with human diagnostic behaviour. Moreover, follow-up predictions still provide significant value in stroke management and patient care. They enable clinicians to evaluate the effectiveness of initial treatments and thereby better predict outcomes or plan additional interventions such as hemicraniectomy.

Since some stroke-related complications may not manifest during the acute phase, follow-up predictions can aid in detecting delayed issues such as cerebral swelling, haemorrhagic transformation or other secondary events. Additionally, tracking lesion progression offers valuable insights for shaping rehabilitation strategies allowing for personalised therapies based on the patient’s evolving condition and recovery potential.

Interpretability of DL models, particularly in the context of medical imaging, is a challenging topic due to the so-called ‘black box’ nature of these models. However, understanding how these models arrive at their decisions is critical for ensuring their reliability and detecting any potential biases.^24^ To address this issue, we employed counterfactual explanations and generated saliency maps that highlight the most relevant parts of the images for our model’s output. Our saliency maps showed that our DL algorithm was able to detect obvious ischaemic lesions with high accuracy while also indicating that the model was less certain about the location of more subtle lesions and may highlight regions outside the true lesion. This behaviour is consistent with that of humans.

Other authors employed a two-stage network to combine local and global information for ischaemic stroke lesion detection^25^ obtaining 87% accuracy. However, in addition to CT scans they also employed Diffusion-weighted imaging (DWI) MR images (which are highly sensitive for early ischaemia and not routinely used in most centres) and their data set is composed of only 277 patients. Mirajkar *et al*^26^ also used a combination of CT and DWI images for the segmentation of stroke lesions. However, our study focuses solely on CT scans and involves a larger-scale investigation to establish a benchmark for this imaging modality. By doing so, we have tried to demonstrate that it is possible to develop future stroke detection algorithms based on routinely acquired (as opposed to optimised for research) CT imaging alone since this is the most widely used imaging modality for acute stroke.

A limitation of our study is that culprit ischaemic lesions may not be visible on CT scans, especially at baseline. This could lead to incorrect labelling of scans. Using healthy controls would have been an option but it is not ethical to scan truly normal individuals with CT due to the associated radiation. While other individuals with ‘normal’ CTs acquired for other reasons may include confounding features. The second limitation is that subgroup analyses exploring the impact of lesion location, lesion number and other chronic features suffer from small numbers of cases in many of the categories.

## Conclusion

Our DL algorithm achieved an accuracy of 72% in detecting the presence of ischaemic lesions and identifying the side of the brain affected by the CT brain scans of patients with stroke symptoms. Our algorithm performed best when lesions were more visible. We found that different lesion types, sizes and chronic brain conditions affected the performance of our system. Our results demonstrate the potential of DL algorithms for detecting ischaemic lesions on CT using a large number of routinely collected scans without lesion annotation. This approach has the potential to develop DL systems from vast numbers of scans, not just those collected for research (as is currently the norm). Such algorithms would much better represent real-life patients with all their natural heterogeneity and ultimately, provide more accurate image interpretation for all patients with acute ischaemic stroke.

## Supplementary material

10.1136/svn-2024-003372 online supplemental file 1

## Data Availability

Data are available in a public, open access repository.
