# Supplementary material for: Development of a deep learning method to identify acute ischaemic stroke lesions on brain CT
Source: Stroke Vasc Neurol. 2024 Nov 20;10(4):e003372. doi: 10.1136/svn-2024-003372 (PMC12415648; doi:10.1136/svn-2024-003372)
Supplement: online supplemental file 1 [file svn-10-4-s001.pdf]

Supplementary Material

Table S1. Hyper-parameters for training the Deep Learning Method.

**Hyper-parameters for half brain model**	
Convolution layer	Convolution2D: kernel size = 3, padding = 1, stride = 1, Filters= [16, 32, 48, 64, 64, 64, 64]
BatchNorm + Leaky ReLU	
Average pool	Averagepool2D: kernel size = 2, padding = 0, stride = 2
Optimiser	Adam, learning rate = 0.001, coseline scheduling, weight decay: 0.00005
**Hyper-parameters for multi-task classifiers**	
Fully connected layer for each task	Task1 FC nodes = 128, Task 2 FC nodes = 128.
Optimiser	Adam, learning rate = 0.0001, coseline scheduling, weight decay: 0.00005
**Hyper-parameters for fine-tuning the entire model**	
Optimiser	Adam, learning rate = 0.00001, coseline scheduling, weight decay: 0.00005

Hyper/parameters employed in our models. The models were trained using a total of eight NVIDIA GeForce RTX 2080 Ti GPUs. Each model is trained for 200 epochs.

Table S2. Accuracy on the test set

	MCA	ACA	PCA	Lacunar	Border zone	Cerebellar	Brain stem
All test scans with lesion region labels (409)	363	28	34	15	7	9	5
Correct classification	248(68%)	21(75%)	18(53%)	5(33%)	6(86%)	3(33%)	1(20%)
Baseline test scans with lesion region labels (148)	135	5	9	4	2	4	0
Correct classification	71(53%)	3(60%)	2(22%)	2(50%)	1(50%)	0(0%)	N/A
Follow-up test scans with lesion region	228	23	25	11	5	5	5

labels (261)							
Correct classification	177(78%)	18(78%)	16(64%)	3(27%)	5(100%)	3(60%)	1(20%)

(a)

		Test scans	Correct classification	Accuracy
1 Lesion	Only MCA	327	216	66%
	Only ACA	7	2	29%
	Only PCA	14	4	29%
	Only lacunar lesion	8	2	25%
	Only cerebellar lesion	7	2	29%
	Only brainstem lesion	4	0	0%
2 Lesions	MCA+ACA	17	15	88%
	MCA+PCA	11	9	82%
	MCA+border zone	2	2	100%
3 Lesions	MCA+ACA+PCA	1	1	100%
	MCA+ACA+lacunar	1	1	100%
	MCA+lacunar+border zone	1	1	100%
	MCA+PCA+border zone	1	1	100%
4 Lesions	MCA+ACA+lacunar+border zone	1	1	100%
5 Lesions	MCA+ACA+PCA+border zone+brain stem	1	1	100%

(b)

	0	1-2	3-4
All test scans with infarct size labels (719)	349	194	176
Correct classification	280(80%)	95(49%)	140(80%)
Baseline test scans (392)	244	77	71
Correct classification	191(78%)	29(38%)	45(63%)
Follow-up (327)	105	117	105
Correct classification	89(85%)	65(56%)	95(90%)

(c)

	Atrophy	Leukoaraiosis	Old stroke lesion	Non-stroke lesion
Scans with other brain conditions (779)	582	398	353	50
AIS lesion (413)	297	196	172	26

No lesion (366)	285	202	181	24
Wrong classification	164(28%)	102(26%)	111(31%)	16(32%)

(d)

Accuracy by lesion location (a), number of lesions (b), infarct size (c) and background conditions (d) on the test set. As expected, the algorithm has better performance when multiple or bigger lesions are present. Old stroke lesions and non-stroke lesions affects classification accuracy the most.

***Table S3*. K-alpha values**

	K-alpha (our algorithm vs each expert)
Expert1	0.2646
Expert2	0.5574
Expert3	0.2895
Expert4	0.3672
Expert5	0.4622
Expert6	0.4622
Expert7	0.4622
Average	0.4093

(a)

	Expert1	Expert 2	Expert 3	Expert 4	Expert 5	Expert 6	Expert 7	Expert consensus	IST-3 label	Our algorithm
Patient1	L	L	L	L	L	L	L	L	L	L
Patient2	N	N	L	N	N	R	N	N	N	N
Patient3	L	L	L	L	L	L	L	L	L	L
Patient4	R	R	R	R	R	R	R	R	R	R
Patient5	L	L	L	L	L	L	L	L	L	L
Patient6	L	L	R	L	L	L	L	L	L	N
Patient7	R	R	R	R	R	R	R	R	N	N
Patient8	L	N	N	R	N	N	N	N	N	N
Patient9	N	N	N	N	N	N	N	N	N	N
Patient10	L	L	L	L	L	N	L	L	L	N
Patient11	R	R	R	R	R	R	R	R	R	R
Patient12	R	N	R	R	R	R	R	R	N	N
Patient13	R	R	B	R	R	R	R	R	R	N

Patient14	L	N	L	N	N	N	N	N	N	N

(b)

Average K-alpha values of our algorithm against each expert (a) and detailed comparison between our algorithm and the 7 experts on the 14 hold-out patients' CT images (b). For patients 7 and 12, the consensus agreement of the experts was different from the clinical gold standard in our dataset, which was matched by our method

Quantitative evaluation of the saliency maps

To evaluate quantitatively how well our MTL model can highlight the areas related to the stroke lesion, we considered a test set of 387 positive scans for which we know the lesion location, which is one of the 6 classes: MCA left, MCA right, ACA left, ACA right, PCA left, PCA right. We registered an arterial atlas of the brain to each scan to locate the different regions and applied gifsplanation. Then, we computed the attribution maps and evaluated them as in previous work [18][19], with the formula:

$$S = \frac{\text{Hits}}{\text{Hits} + \text{Misses}}$$

A hit is counted if the voxel with the greatest change lies in the correct region, a miss is counted otherwise.

We obtain a score of 58.25 on our test set. As we observed when discussing the classification accuracy of our model, small and very small lesions (infarct size 1 or 2) are more difficult to detect, resulting in a score of 48.86. On the other hand, medium and big lesions (size 3-4) obtain a higher score of 70.28.

Figures

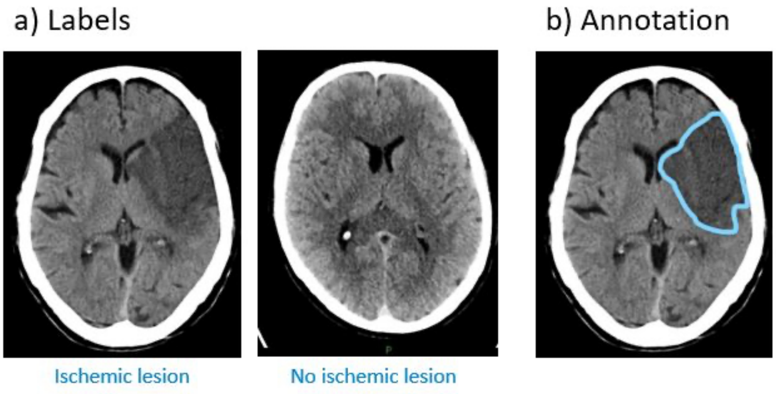

Figure S1. Difference between labels (a) and annotation (b). Our data included the former but not the latter.

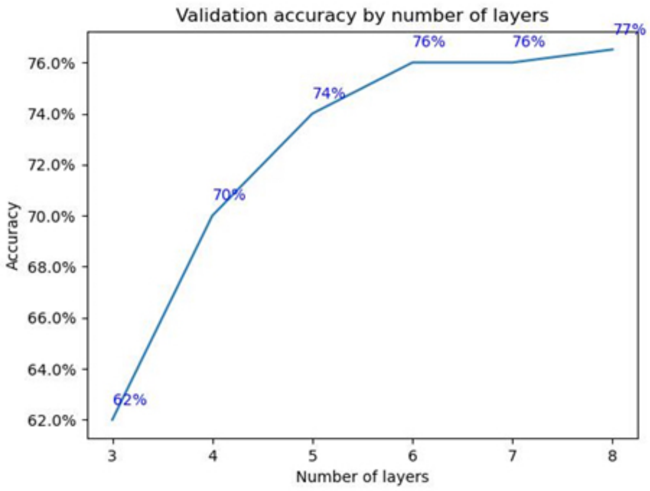

Figure S2. Validation accuracy by number of convolutional layers.
